# Impact of maternal education on the outcome of newborns requiring surgery for congenital malformations

**DOI:** 10.1371/journal.pone.0214967

**Published:** 2019-04-08

**Authors:** Carmen Dingemann, Martin Sonne, Benno Ure, Bettina Bohnhorst, Constantin von Kaisenberg, Sabine Pirr

**Affiliations:** 1 Department of Pediatric Surgery, Hannover Medical School, Hannover, Germany; 2 Department of Pediatric Pulmonology, Allergology and Neonatology, Hannover Medical School, Hannover, Germany; 3 Department of Obstetrics, Gynecology and Reproductive Medicine, Hannover Medical School, Hannover, Germany; Stony Brook University Health Sciences Center School of Medicine, UNITED STATES

## Abstract

**Objective:**

Numerous studies established a link between socioeconomic status (SES) and several dimensions of general health. This study examines the association between maternal education as a widely used indicator of SES and outcome in newborns requiring surgical correction of congenital anomalies.

**Methods:**

Ambispective data analysis of newborns with esophageal atresia (EA), intestinal atresia (IA), congenital diaphragmatic hernia (CDH), omphalocele (OC), gastroschisis (GS) undergoing surgery between 01/2008-11/2017 accessing the clinical databases Neodat and Viewpoint. Maternal education was determined according to the validated education classification CASMIN and stratified into “low” SES and “high” SES group. Endpoints were incidence of postoperative complications, length of mechanical ventilation, and readmission to NICU.

**Results:**

Inclusion of 169 patients with EA (n = 32), IA (n = 24), CDH (n = 47), OC (n = 19), GS (n = 47). Women of low SES (n = 67, 40%) attended fewer prenatal screenings (total, 4.6 vs. 7.9, P<0.0001; EA, 3.7 vs. 7.1, P = 0.0002; IA, 3.5 vs. 9.4, P = 0.0006; OC, 2.5 vs. 8.8, P = 0.009; GS, 4.1 vs. 7.0, P = 0.002). Low SES was associated with higher incidence of patients born small for gestational age (37% vs. 20%, P = 0.019), with additional congenital malformations (37% vs. 15%, P = 0.001), being born in a peripheral center (7% vs. 0%, P = 0.008), and with higher incidence of 5´APGAR scores <7 (23% vs. 7%, P = 0.004). Moreover, low SES was associated with higher incidence of postoperative complications (total 70% vs. 32%, P<0.0001; EA, 60% vs. 23%, P = 0.04; IA, 67% vs. 11%, P = 0.008; CDH, 83% vs. 46%, P = 0.009; GS, 74% vs. 25%, P = 0.001), and higher readmission rate to NICU (IA, 33% vs. 0%, P = 0.043; GS, 32% vs. 4%, P = 0.007).

**Conclusions:**

Low maternal education is associated with a reduced uptake of prenatal screenings, adverse neonatal outcomes, and higher incidence of postoperative complications in newborns with congenital anomalies. Primary prevention and specific support should be provided prenatally for families with low SES to avoid adverse outcomes.

## Introduction

The Member States of the World Health Organization have constituted universal health insurance coverage as an important goal in the development of health financing systems [1]. In common with other countries, the German health service provides universal coverage for healthcare, including obstetric, neonatal and related health care services to women, regardless of their socioeconomic status (SES), race or ethnicity. Therefore, neonatal outcomes are expected not to be affected by socioeconomic inequalities in health systems with universal access to essential health services [2].

However, epidemiological studies have indicated an association between socioeconomic factors and several dimensions of general health [3, 4, 5, 6], with increases in SES being associated with striking benefits to health [5, 6]. SES-based disparities have been demonstrated across a range of health outcomes in adults, including morbidity such as cardiovascular disease [7], diabetes [8], and overall mortality [9].

In the neonatal population, different SES measures capture unique aspects and pathways of socioeconomic disparities that can relate differently to child health [10, 11]. Among maternal social aspects, maternal education is considered the most powerful determinant of health [12] and the most frequently reported indicator of SES [13].

Numerous studies have linked maternal socioeconomic disadvantage with adverse neonatal and developmental outcomes, as premature birth and different aspects of cognitive and developmental delay [10, 13, 14, 15, 16, 17, 18, 19].

In contrast, literature provides little evidence on the impact of maternal SES on the outcome of neonates undergoing surgery for congenital malformations, such as esophageal atresia (EA), intestinal atresia (IA), congenital diaphragmatic hernia (CDH), omphalocele (OC), and gastroschisis (GS) [20, 21].

Common to these congenital malformations is their need for prompt surgical correction within the first days of life and intensive neonatal care. The operative management of patients with EA aims to restore the interruption of the esophageal continuity by an anastomosis of both blind-ended pouches to allow a normal gastrointestinal passage. The same surgical principle applies to patients with IA including duodenal atresia/stenosis and small bowel atresia/stenosis. CDH is characterized by a diaphragmatic defect along with pulmonary hypoplasia. After preoperative stabilization of the patient, the surgical management consists of the closure of the diaphragm. Both OC and GS are congenital abdominal wall defects. The general principle of surgical management of the two conditions consists of closure of the abdominal wall defect, while minimizing the risk of injury to the abdominal viscera.

Aim of this study was to investigate the association between maternal education and outcome in newborns requiring prompt surgical correction of congenital malformations.

## Patients and methods

This study was approved by the Institutional Ethical Review Board of Hannover Medical School, Carl-Neuberg-Str. 1, 30625 Hannover, Germany (approval number 3666–2017). Written informed consent was obtained from all guardians for anonymized data analysis and publication. We performed an ambispective data analysis of newborns with isolated congenital malformations of EA, IA, CDH, OC, and GS. This ambispective study is characterized by both *retrospective* (data analysis obtained from databases) and *prospective* (systematic interviews of parents) components. This research includes all patients that have been admitted to our neonatal intensive care unit (NICU), whether they were born in-house or have been referred by another hospital for further treatment. All included patients underwent primary surgical correction in our tertiary referral center between 1^st^ January 2008 and 30^th^ November 2017.

**Fig 1:**
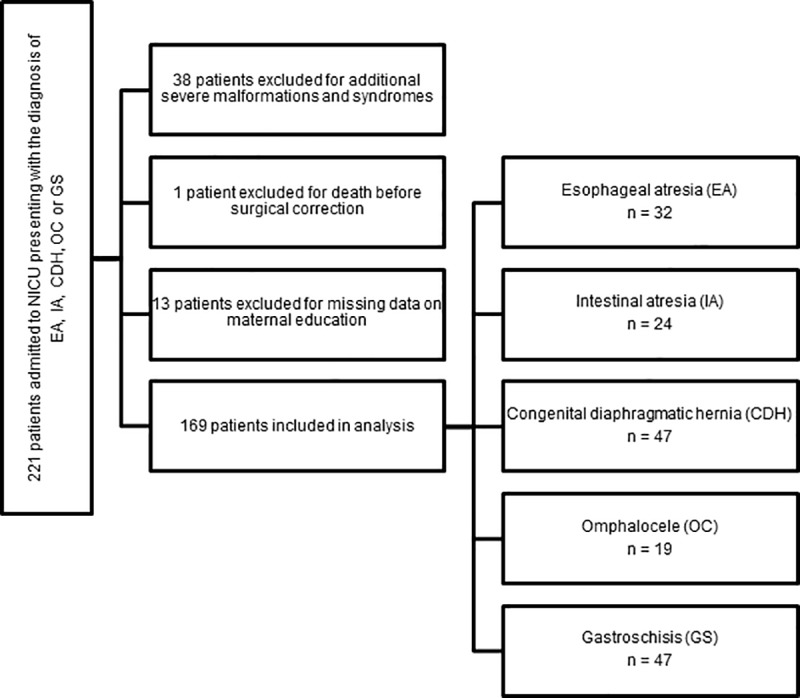
Flow diagram of patient recruitment and number of included patients between 01/2008 and 11/2017.

Patients with additional relevant malformations and syndromes, i.e. severe cardiac, genetic or urogenital anomalies requiring further interventions during the initial hospital stay have been excluded. Patient recruitment and number of included patients are shown in Fig 1.

Sociodemographic and clinical data were obtained from the databases *Neodat* and *Viewpoint*, and were analyzed anonymously. *Neodat* is a modular neonatal-pediatric patient database system for integrated data acquisition for quality assurance, medical reports and forms, documentation of screenings and follow-up as well as integrated clinical and cross-departmental modules. In order to obtain a complete record of all relevant data including information on the maternal educational attainment, the data collection system of the Department of Gynecology and Obstetrics *Viewpoint* was accessed in addition. In case of missing data, the patients´ parents were contacted via telephone interviews in order to complete the database.

Endpoints of this study were incidence of postoperative complications, length of perioperative mechanical ventilation, and readmission to the NICU after transfer to an in-house peripheral ward during the initial hospital stay. Postoperative complications summarized pooled complications not requiring any surgical re-intervention (such as wound infection, vena cava thrombosis, chylothorax, pleural effusion and pneumothorax), sepsis, surgical revision, and mortality. Furthermore, postoperative complications were classified in six categories that are consistent with grade II to grade V of the Clavien-Dindo Classification [22] and analyzed for number and severity. In case a patient presented with more than one postoperative complication, the one with the highest grading according to the Clavien-Dindo Classification was entered in the severity analysis. Readmission to the NICU was defined as readmission for various reasons from a general pediatric in-house ward during the initial hospital stay.

### Social stratification

In order to determine maternal education, the CASMIN (Comparative Analysis of Social Mobility in Industrial Nations) Educational Classification [23, 24, 25, 26] as an international comparable measurement instrument for educational attainment was used. This classification is a standard approach to record educational level in social science. Educational attainment is a widely used indicator of SES in health studies [6, 26, 27]. The use of education based cut-offs represents a simple, clinically applicable decision rule. Furthermore, questions about education are less private than questions about family income and are more reliably reported [26, 28]. The CASMIN Educational Classification is applicable in various countries and allows comparison of our findings with a wider range of studies [25, 26, 29]. Moreover, it is based upon two primary classification criteria: 1) the differentiation of a hierarchy of educational levels, both in terms of the length of the educational experience as well as in the required intellectual abilities and corresponding curricular contents, and 2) the differentiation between”general” and”vocationally-oriented” education [23, 25]. The CASMIN Educational Classification consists of a coding schema containing nine levels of educational qualification [23, 26, 29]. These nine levels can be hierarchical divided and equally well taken as ordering positions in terms of the SES [26].

In this study, the original nine levels have been merged into the two groups *Low Educational Level (LEL)* referring to “low SES” and *High Educational Level (HEL)* referring to “high SES”. For CASMIN levels and allocation to the two groups see Table 1.

**Table 1:** CASMIN Educational Classification levels and modified allocation into two groups.

CASMIN 1a	Inadequately completed elementary education	LEL low SES
CASMIN 1b	Completed elementary education	
CASMIN 1c	Elementary education and basic vocational qualification	
CASMIN 2b	Intermediate general qualification *without* vocational qualification	
CASMIN 2a	Intermediate general qualification *and* vocational qualification	HEL high SES
CASMIN 2c- *gen*	Full maturity certificates *without* vocational qualification	
CASMIN 2c- *voc*	Full maturity certificates *with* vocational qualification	
CASMIN 3a	Lower tertiary education (technical college diplomas)	
CASMIN 3b	Higher tertiary education (university teaching certificates)	

CASMIN Educational Classification as per [25]

LEL—low educational level; HEL—high educational level

### Statistical analysis and software

All data were analyzed in an anonymized form. Incomplete datasets were excluded from statistical analysis (n = 13, see Fig 1). Pre- and perinatal data were analyzed jointly for all included patients. Due to the marked diversity and complexity of the included congenital malformations and resulting individual course, data on postoperative outcome were analyzed separately for each patient group.

Means and standard deviations were calculated for continuous variables, and frequencies and percentages for categorical variables. Differences in demographic and clinical characteristics were assessed using Student's *t*-test and chi-square test where appropriate. The degree of correlation between variables was analyzed using Pearson’s correlation. Data were tested for normal distribution and equality of variances. Data management and statistical analyses were realized with Excel 2010 (Microsoft Corporation, Redmond, WA, USA) and SPSS (version 25; SAS Institute, Cary, NC, USA). Statistical significance was set at the 0.05 level.

## Results

### Patient characteristics

In total, 169 infants with congenital malformations requiring prompt surgical intervention have been included in this study. Patient distribution to EA, IA, CDH, OC and GS is shown in Fig 1. Patients have been allocated into LEL and HEL according to maternal educational attainment. As per definition in this study, LEL refers to low maternal SES, whereas HEL represents high maternal SES. Table 2 shows the distribution of maternal SES for each congenital malformation.

**Table 2:** Distribution of maternal socioeconomic status (SES) of newborns with a congenital malformation requiring surgical intervention as determined according to the modified CASMIN Educational Classification.

	Esophageal atresia n (%)	Intestinal atresia n (%)	Congenital diaphragmatic hernia n (%)	Omphalocele n (%)	Gastroschisis n (%)	Total n (%)
LEL	10 (31)	11 (46)	23 (49)	4 (21)	19 (40)	67 (40)
HEL	22 (69)	13 (54)	24 (51)	15 (79)	28 (60)	102 (60)
Total	32	24	47	19	47	169

LEL corresponds to a low maternal SES; HEL corresponds to a high maternal SES.

Over all patients, there were n = 67 (40%) mothers with a LEL background and n = 102 (60%) mothers with a HEL background. The majority of patients with EA (69% vs. 31%; P = 0.08), OC (79% vs. 21%; P = 0.036) and GS (60% vs. 40%; P = 0.28) presented with a high SES.

Demographic data on patients are presented in Table 3.

**Table 3:** Characteristics of n = 169 included patients with congenital malformation.

Congenital malformation	Patient characteristics	LEL	HEL	P value
EA	Sex; male	7 (70)	14 (64)	0.725
	Gestational age; weeks	36.0 (4.1)	34.7 (3.6)	0.369
	Age of mother at birth; years	30 (6.5)	34 (5.5)	0.086
	Parity	2.0 (1.1)	1.7 (1.1)	0.509
	Delivery by Cesarean section	6 (60)	13 (59)	0.963
	Inborn	3 (30)	7 (32)	0.921
	Age at admission to NICU; days	0.2 (0.4)	0.2 (0.7)	0.909
IA	Sex; male	9 (60)	7 (78)	0.393
	Gestational age; weeks	35.1 (3.5)	35.6 (3.2)	0.737
	Age of mother at birth; years	28 (6.8)	32 (4.5)	0.095
	Parity	1.9 (1.1)	1.8 (0.4)	0.814
	Delivery by Cesarean section	4 (27)	6 (67)	0.058
	Inborn	9 (60)	6 (67)	0.757
	Age at admission to NICU; days	0.5 (1.1)	1.6 (3.4)	0.265
CDH	Sex; male	11 (48)	12 (50)	0.882
	Gestational age; weeks	38.5 (2.0)	38.2 (2.1)	0.545
	Age of mother at birth; years	27 (6.1)	30 (5.7)	0.088
	Parity	2.0 (1.4)	1.5 (0.9)	0.115
	Delivery by Cesarean section	13 (57)	14 (58)	0.903
	Inborn	13 (57)	11 (46)	0.475
	Age at admission to NICU; days	0.04 (0.2)	6.3 (14.7)	**0.0493**
OC	Sex; male	2 (50)	9 (60)	0.719
	Gestational age; weeks	38.1 (0.5)	36.7 (1.7)	0.129
	Age of mother at birth; years	24 (4.6)	32 (4.8)	**0.0078**
	Parity	2.0 (0.8)	1.5 (0.5)	0.121
	Delivery by Cesarean section	4 (100)	13 (87)	0.468
	Inborn	4 (100)	15 (100)	n.a.
	Age at admission to NICU; days	0.0 (0)	0.0 (0)	n.a.
GS	Sex; male	8 (42)	12 (43)	0.959
	Gestational age; weeks	35.8 (1.8)	35.5 (2.0)	0.635
	Age of mother at birth; years	23 (4.6)	29 (5.3)	**0.0002**
	Parity	1.7 (0.9)	1.3 (0.5)	**0.0395**
	Delivery by Cesarean section	19 (100)	28 (100)	n.a.
	Inborn	18 (95)	28 (100)	0.229
	Age at admission to NICU; days	0.0 (0)	0.0 (0)	n.a.

Data for sex, mode of delivery and inborn patients are given as number and percentage. Data for gestational age, age of mother at birth, parity and age of the patient at admission are given as mean and standard deviation. P values were determined either by Student’s *t*-test or chi-square test as applicable. Significant differences are in bold.

### Postoperative outcome

A low maternal education was associated with a significantly higher incidence of postoperative complications in the whole collective of patients (70% LEL vs. 32% HEL, P < 0.0001, OR 5.1, 95% CI 2.6–10.0). This finding became also evident in the group of patients with EA (60% LEL vs. 23% HEL, P = 0.04; OR 5.1, 95% CI 1.0–25.5), with IA (67% LEL vs. 11% HEL, P = 0.008, OR 8.0, 95% CI 1.2–52.7), with CDH (83% LEL vs. 46% HEL, P = 0.009, OR 5.6, 95% CI 1.5–21.5), and with GS (74% LEL vs. 25% HEL, P = 0.001, OR 8.4, 95% CI 2.2–31.8), but not in patients with OC (25% LEL vs. 47% HEL, P = 0.435).

Furthermore, in IA, CDH and GS patients with a low SES background, postoperative complications were of higher severity compared to patients with a high SES as measured by the Clavien-Dindo Classification. Postoperative complications are specified in accordance with the Clavien-Dindo Classification in Table 4.

**Table 4:** Definition of postoperative complications and categorization according to the Clavien-Dindo Classification.

Congenital malformation	Clavien-Dindo-Classification	Number of patients with postoperative complication n		Specification (n)
		LEL	HEL	
EA	II	1	0	sepsis (1)
	IIIa	2	0	chylothorax (1), pneumothorax (1)
	IIIb	3	5	surgical revision^a^ (4), anastomotic stricture (4)
	IVa	0	0	
	IVb	0	0	
	V	0	0	
	P value severity	0.1597		
IA	II	5	0	sepsis (4), wound infection (1)
	IIIa	0	0	
	IIIb	5	1	surgical revision^b^ (5), incisional hernia (1)
	IVa	0	0	
	IVb	0	0	
	V	0	0	
	P value severity	**0.0008**		
CDH	II	3	1	sepsis (2), thrombosis (2)
	IIIa	4	3	chylothorax (6), pleural effusion (1)
	IIIb	2	4	surgical revision^c^ (6)
	IVa	1	1	reentry-tachycardia (1), sepsis with single organ failure (1)
	IVb	2	0	sepsis with multiple organ failure (1), CPR due to hemorrhage (1)
	V	7	2	death (9)
	P value severity	**0.0091**		
OC	II	1	1	sepsis (2)
	IIIa	0	0	
	IIIb	0	6	abdominal wall hernia (3), surgical revision^d^ (2), incisional hernia (1)
	IVa	0	0	
	IVb	0	0	
	V	0	0	
	P value severity	0.2032		
GS	II	5	5	sepsis (10)
	IIIa	0	0	
	IIIb	5	0	surgical revision^e^ (5)
	IVa	2	1	sepsis with single organ failure (3)
	IVb	1	0	sepsis with multiple organ failure (1)
	V	1	1	death (2)
	P value severity	**0.0001**		

P values were determined by Student’s t-test. Significant differences are in bold. P values were determined by Student’s *t*-test. Significant differences are in bold.

Surgical revision was done for (n)

^a^fundoplicatio (1), adhesiolysis and re-formation of gastrostomy (1), formation of jejunostomy for feeding issues (1) and pyloroplasty for refractory gastroparesis (1)

^b^anastomotic leakage (1), mechanical ileus (1), second intestinal atresia (1), intraabdominal bleeding (1) and serial transverse enteroplasty for short bowel sydrome (1)

^c^recurrence (2), adhesiolysis (2), closure of hiatal hernia (1) and wound revision and adaptation (1)

^d^adhesiolysis (1) and abdominal compartment (1)

^e^intestinal atresia (2), intestinal necrosis (1), abscess drainage (1) and serial transverse enteroplasty for short bowel syndrome (1)

With regard to length of perioperative mechanical ventilation, there were no significant differences between LEL and HEL in any of the patient groups (mean duration of mechanical ventilation in days for LEL vs. HEL in each group: EA 11.0 vs. 5.6, P = 0.151; IA 4.1 vs. 2.0, P = 0.598; CDH 18.0 vs. 18.2, P = 0.972; OC 0.3 vs. 3.2, P = 0.190; GS 3.7 vs. 4.8, P = 0.542).

**Fig 2:**
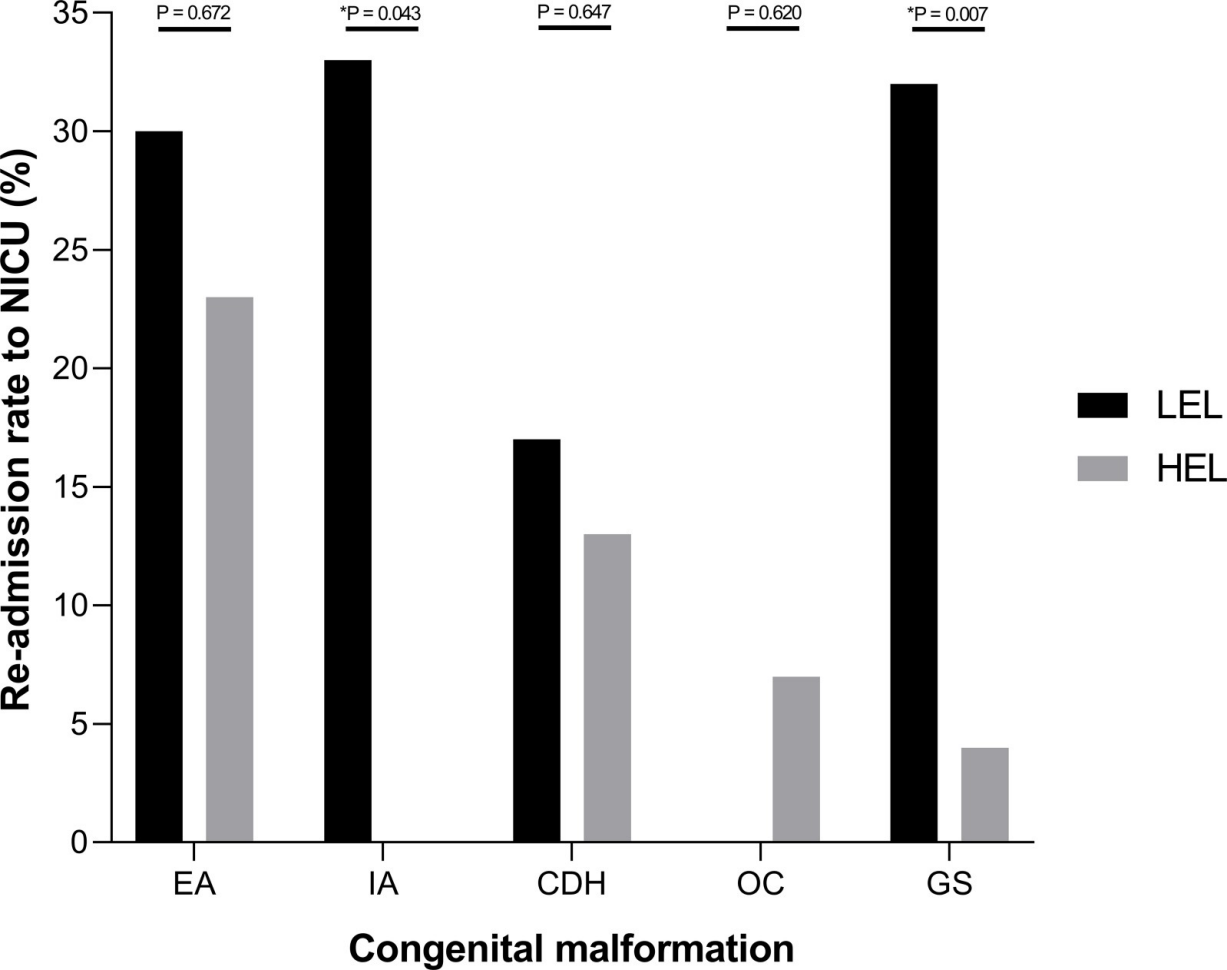
Re-admission rate to NICU. Percentage stratified for the different patient groups, P-values were determined by Student’s *t*-test.

A low socioeconomic background in patients with IA (33% LEL vs. 0% HEL; P = 0.043) and GS (32% LEL vs. 4% HEL; P = 0.007) was associated with a higher readmission rate to the NICU due to either surgical revision under general anesthesia, sepsis with organ failure or respiratory deterioration. There were no differences between LEL and HEL in patients with EA, CDH, or OC (Fig 2).

### Perinatal conditions and neonatal outcome

All patients received uniform neonatal and surgical treatment regardless of their socioeconomic background. Therefore, pre- and perinatal conditions have also been assessed in order to identify potential factors affecting their outcome.

**Fig 3:**
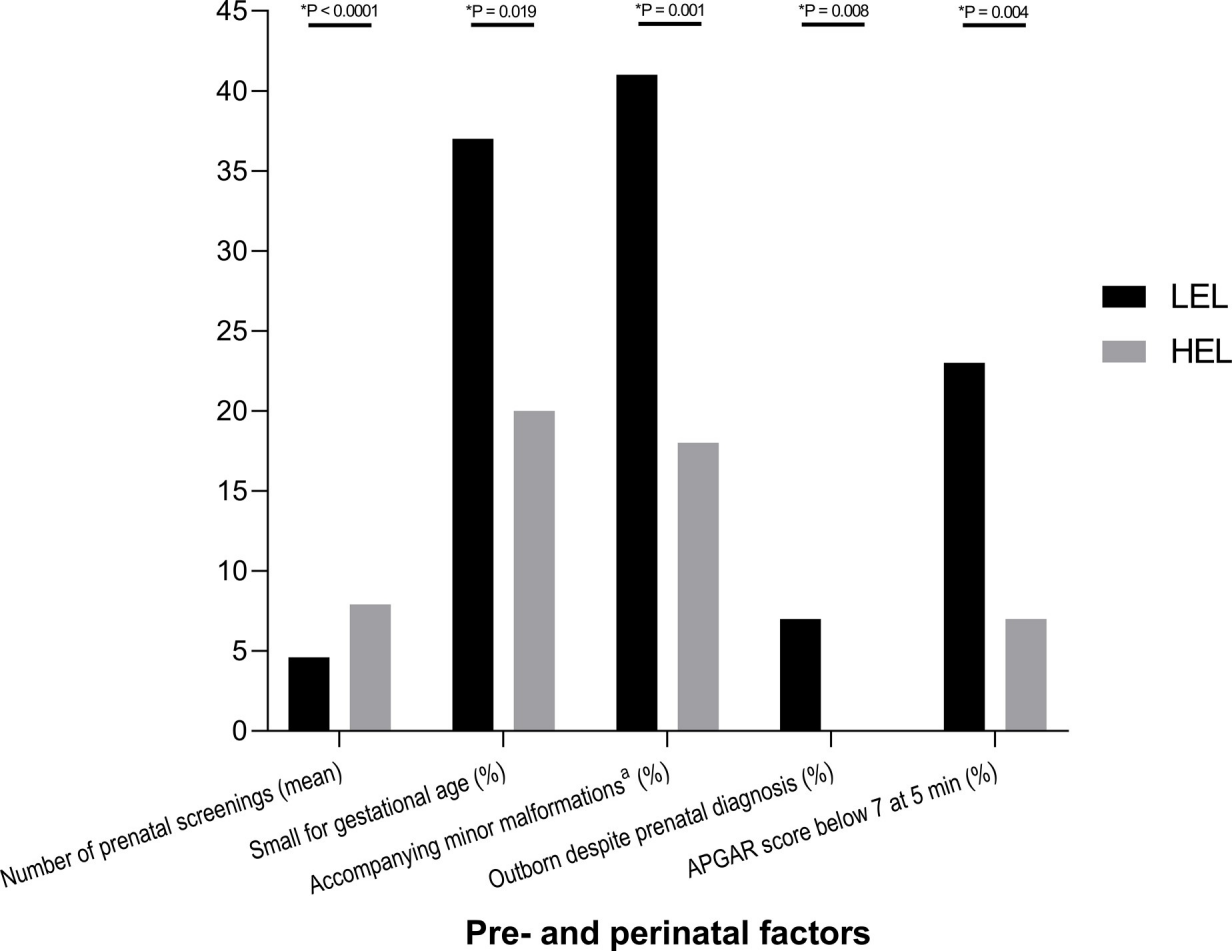
Pre- and perinatal conditions in patients with congenital malformations requiring surgical intervention. Data are shown as mean and standard deviation or percentage as indicated. P values were determined either by Student’s t-test or chi-square test as applicable. ^a^minor malformations were defined as malformations and syndromes that did not require any additional intervention during the initial hospital stay, such as atrial or ventricular septal defect, dextrocardia, aplasia of the inferior vena cava with persistent azygos vein, truncus bicaroticus, trisomy 21 without cardiac defects except atrial or ventricular septal defects, Wiedemann-Beckwith-Syndrome, intestinal mal- or nonrotation, horseshoe or pelvic kidney, renal duplication, megaureter, hypospadia, polysplenia, butterfly vertebrae, cleft palate, unilateral vocal cord paralysis, septum pellucidum agenesis, and corpus callosum hypoplasia.

Overall, mothers with a LEL background attended significantly fewer prenatal screenings than mothers with a HEL background (4.6 LEL total vs. 7.9 HEL total, P < 0.0001), (Fig 3).

These findings became evident for mothers of patients with EA (number of prenatal screenings 3.7 LEL vs. 7.1 HEL, P = 0.0002), with IA (3.5 LEL vs. 9.4 HEL, P = 0.0006), with OC (2.5 LEL vs. 8.8 HEL, P = 0.009), and with GS (4.1 LEL vs. 7.0 HEL, P = 0.002). Even though the result for the group of CDH patients did not reach the level of significance (number of prenatal screenings 6.7 LEL vs. 8.7 HEL, P = 0.09), there was a clear trend towards decreased uptake of screening services by women with a low educational background.

Low maternal SES was associated with higher incidence of patients born small for gestational age (37% LEL vs. 20% HEL, P = 0.019), higher incidence of additional minor congenital malformations (37% LEL vs. 15% HEL, P = 0.001), higher proportion of patients born in a peripheral hospital despite prenatal diagnosis (7% LEL vs. 0% HEL, P = 0.008), and higher incidence of APGAR scores below 7 at 5 minutes postnatally (23% LEL vs. 7% HEL, P = 0.004), (Fig 3).

Furthermore, we analyzed the incidence of premature birth in LEL and HEL, but did not find a significant difference in our cohort (46% LEL vs. 57% HEL, P = 0.173).

In addition, potential correlations among these perinatal factors themselves have been analyzed. This analysis revealed that a higher number of prenatal screenings correlated with a more frequent prenatal diagnosis (r = 0.21; P = 0.0007), a lower incidence of postoperative complications (r = -0.29; P = 0.0002), a lower incidence of sepsis (r = -0.27; P = 0.0005), and a lower readmission rate to the NICU (r = -0.23; P = 0.003). Moreover, it could be demonstrated that the higher the APGAR scores at 5 minutes postnatally, the lower the incidence of postoperative complications (r = -0.18; P = 0.02), and the lower the mortality rate (r = -0.28; P = 0.0002). Patients born small for gestational age were at higher risk of getting readmitted to the NICU (r = 0.16; P = 0.04). It could also be shown that patients born in a peripheral hospital presented with a significantly higher incidence of postoperative complications (r = 0.18; P = 0.02), a higher readmission rate to the NICU (r = 0.18; P = 0.02), and a higher mortality rate (r = 0.38; P < 0.0001).

## Discussion

Educational attainment is a widely used indicator of SES in health studies [6, 27]. The use of education based cut-offs represents a simple, clinically applicable decision rule. Questions about education are less private than questions about family income and are more reliably reported [28]. According to a systematic review of studies in industrialized countries, maternal education, rather than maternal income, has been found to correlate with birth outcomes [18]. Therefore, it has been decided to use maternal educational level as a proxy for social stratification in this study.

Several studies have demonstrated that maternal SES strongly affects child health which in part can be attributed to differences in attendance of prenatal care and adverse birth outcomes [10, 11, 30, 31, 32, 33]. Moreover, Joseph et al. stated that differences in the ability to access good-quality obstetric services and neonatal care may be due to differences in maternal SES [2]. It has been postulated that disparate prenatal uptake rates in a group of socioeconomically diverse women could be explained by their individual attitudes and perceptions of burden and value of information [34, 35]. Knowledge of screening and levels of informed choice have been shown to be higher in socioeconomically advantaged women [32, 33, 36].

The findings of our study are highly consistent with the above reported results as we could demonstrate that mothers with low socioeconomic background attended significantly fewer prenatal screenings than mothers with high SES.

In addition, it is widely accepted that maternal factors which are attributed to SES, such as drug use, cigarette smoking during pregnancy and nutrition are responsible for negative neonatal outcomes [37, 38]. Although we did not investigate these factors attributed to SES, our data clearly show that low maternal educational setting is associated with adverse birth outcomes.

Health disparities are the result of complex, multilevel, dynamic factors, including biological, environmental, and social elements [20]. Recent pediatric surgical literature has identified racial disparities in pediatric surgical outcomes, even after controlling for established patient- and hospital-related factors [20]. Stone et al., when evaluating postoperative morbidity, mortality, and resource utilization in several pediatric surgeries identified risk-adjusted associations of race with poorer outcomes and higher resource utilization [39]. Although these publications report on the correlation between race and pediatric surgical outcomes, race is closely linked to SES [40]. For this reason, the findings of the reported studies are in line with our current data. Prior studies have identified physician–patient communication, provider bias, resource allocation, access to prenatal care, access to specialized care and birth at a pediatric hospital as influencing factors in pediatric surgical outcomes [41, 42, 43].

Notwithstanding, literature is scarce on the potential impact of maternal education on congenital malformations requiring surgical intervention, such as EA, IA, CDH, OC, and GS [20, 21, 44, 45]. The vast majority of studies concentrated on potential correlations between socioeconomic background and the condition´s etiology: It has been postulated that low maternal SES, among other factors, is a potential risk factor in the origin of EA [44, 46]. Chircor et al. stated that maternal SES might be a risk factor in the etiology of OC and GS [45]. Mastroiacovo et al. postulated that low maternal SES is one of several characteristics (such as drug consumption during pregnancy) common to mothers of neonates born with GS [47]. Significant associations between the occurrence of GS and low SES have been described elsewhere [48, 49].

Only few studies focused on the postnatal and postoperative outcome of patients with congenital malformations: Stolar et al. demonstrated that maternal educational status is an important predictor of neurodevelopmental outcome in children with CDH [21]. These findings have been confirmed by others [50, 51].

Song et al. investigated outcomes of 3846 neonates with GS [20]. They could demonstrate that parental income status is associated with mortality and hospital charges while payer status is associated with complications, mortality, lengths of stay, and hospital charges [20]. Their data suggested that social factors (more than biologic determinants) associated with low SES, such as a lack of adequate prenatal care, may contribute to the poorer outcomes observed in these groups [20].

This hypothesis is strongly supported by the results of our study as we clearly demonstrate the existence of a social gradient in the outcome of newborns with congenital malformations requiring prompt surgical management. Out data suggest that, in a setting where the healthcare system provides universal health services to all women, irrespective of their SES, low maternal education level is strongly associated with some adverse neonatal outcomes, including worse clinical condition, increased number of infants born small for gestational age, and increased number of associated malformations. Moreover, maternal socioeconomic factors contribute to adverse postoperative outcomes, including increased number of postoperative complications and higher readmission rate to NICU.

It is tempting to speculate that the mother´s pre-pregnancy and prenatal behavior might be responsible for both the neonatal and resultant postoperative outcome. This study indicates that serious prenatal failures cannot be compensated postnatally. Based on the presented data, it can only be stated that a low socioeconomic background of neonates is associated with negative preconditions in comparison to neonates with a high socioeconomic setting.

### Limitations

First, the low number of included patients is one major problem affecting the quality of the present study. However, the low incidence of the included congenital malformations varying from 1:2.500 (EA) to 10.000 (IA) live births may explain the presented figures.

Second, it could be demonstrated that maternal socioeconomic factors contributed to adverse postoperative outcomes in many cases investigated, but not in all cases. The authors hypothesize that this is attributable to the described marked diversity and complexity of included congenital malformations resulting in individual courses and outcomes.

Third, it was omitted to conduct a sample size calculation as the study period already amounts to 11 years. The authors believe that a more extended period might have caused bias due to modifications of treatment.

Fourth, it was not possible to collect further information on mothers of included patients, such as annual household income, marital status, underlying disease, etc. These are factors which have been recognized to be associated with perinatal outcomes in previous studies [2, 11].

Finally, the lack of data on important maternal factors, such as cigarette smoking, drug consumption, pre-pregnancy weight and gestational weight gain [10, 37, 38], may further contribute to some unavoidable source of systematic uncertainty.

### Conclusions

Even in a country with access to universal health care services, low maternal education is associated with a reduced uptake of prenatal screening, poorer neonatal outcomes, and a higher incidence of postoperative complications in newborns with congenital malformations.

These results should provide the basis for future studies investigating factors mediating the effect of socioeconomic inequality on postnatal outcomes. In order to reduce current social inequalities, specific support should be provided especially prenatally for families with low socioeconomic background. Prospective strategies are vital to improve perinatal healthcare and targeted perinatal intervention to avoid adverse outcomes.

## Supporting information

S1 Table The study’s underlying data set. (XLSX)

## References

[1] Carrin G, Mathauer I, Xu K, Evans DB. Universal coverage of health services: tailoring its implementation. Bull World Health Organ. 2008;86:857–63. 10.2471/BLT.07.049387 19030691 PMC2649543

[2] Joseph K, Liston R, Dodds L, Dahlgren L, Allen A. Socioeconomic status and perinatal outcomes in a setting with universal access to essential health care services. CMAJ. 2007;177:583–90. 10.1503/cmaj.061198 17846440 PMC1963370

[3] Gößwald A, Lange M, Dölle R, Hölling H. The first wave of the German Health Interview and Examination Survey for Adults (DEGS1): participant recruitment, fieldwork, and quality management. Bundesgesundheitsblatt Gesundheitsforschung Gesundheitsschutz. 2013;56(5–6):611–9. 10.1007/s00103-013-1671-z 23703477

[4] Alvarez-Galvez J, Rodero-Cosano ML, Motrico E, Salinas-Perez JA, Garcia-Alonso C, Salvador-Carulla L. The impact of socio-economic status on self-rated health: study of 29 countries using European social surveys (2002–2008). Int J Environ Res Public Health. 2013;10:747–61. 10.3390/ijerph10030747 23439514 PMC3709282

[5] Adler NE, Newman K. Socioeconomic disparities in health: pathways and policies. Health Affairs. 2002;21(2):60–76. 10.1377/hlthaff.21.2.60 11900187

[6] Hardarson T, Gardarsdóttir M, Gudmundsson KT, Thorgeirsson G, Sigvaldason H, Sigfússon N. The relationship between educational level and mortality. The Reykjavík Study. J Intern Med. 2001;249(6):495–502. 11422655 10.1046/j.1365-2796.2001.00834.x

[7] Kaplan GA, Keil JE. Socioeconomic factors and cardiovascular disease: a review of the literature. Circulation. 1993;88(4 Pt 1):1973–1998. 8403348 10.1161/01.cir.88.4.1973

[8] Everson SA, Maty SC, Lynch JW, Kaplan GA. Epidemiologic evidence for the relation between socioeconomic status and depression, obesity, and diabetes. Journal of Psychosomatic Research. 2002;53(4):891–895. 12377299 10.1016/s0022-3999(02)00303-3

[9] Feinglass J, Lin S, Thompson J, Sudano J, Dunlop D, Song J, et al Baseline health, socioeconomic status, and 10-year mortality among older middle-aged Americans: findings from the Health and Retirement Study, 1992–2002. Journals of Gerontology Series B-Psychological Sciences & Social Sciences. 2007;62(4):S209–17. 10.1093/geronb/62.4.s209 17673534

[10] Cantarutti A, Franchi M, Monzio Compagnoni M, Merlino L, Corrao G. Mother's education and the risk of several neonatal outcomes: an evidence from an Italian population-based study. BMC Pregnancy Childbirth. 2017;17(1):221 10.1186/s12884-017-1418-1 28701151 PMC5508478

[11] Mortensen HL, Helweg-Larsen K, Andersen AMN. Socioeconomic differences in perinatal health and disease. Scand J Public Health. 2011;39(Suppl 7):110–4. 21775367 10.1177/1403494811405096

[12] Luo ZC, Wilkins R, Kramer MS, Fetal and Infant Health Study Group of the Canadian Perinatal Surveillance System. Effect of neighbourhood income and maternal education on birth outcomes: a population-based study. CMAJ. 2006;174(10):1415–20. 10.1503/cmaj.051096 16682708 PMC1455422

[13] Wong HS, Edwards P. Nature or nurture: a systematic review of the effect of socio-economic status on the developmental and cognitive outcomes of children born preterm. Matern Child Health J. 2013;17(9):1689–700. 10.1007/s10995-012-1183-8 23135625

[14] Potijk MR, Kerstjens JM, Bos AF, Reijneveld SA, de Winter AF. Developmental delay in moderately preterm-born children with low socioeconomic status: risks multiply. J Pediatr. 2013;163(5):1289–95. 10.1016/j.jpeds.2013.07.001 23968750

[15] Wild KT, Betancourt LM, Brodsky NL, Hurt H. The effect of socioeconomic status on the language outcome of preterm infants at toddler age. Early Hum Dev. 2013;89(9):743–6. 10.1016/j.earlhumdev.2013.05.008 23803578

[16] Krägeloh-Mann I, Lidzba K. Preterm cognitive outcome and socioeconomic status. Acta Paediatr. 2012;101(6):557–8. 10.1111/j.1651-2227.2012.02622.x 22296570

[17] Tong S, Baghurst P, Vimpani G, McMichael A. Socioeconomic position, maternal IQ, home environment, and cognitive development. J Pediatr. 2007;151(3):284–8, 288.e1. 10.1016/j.jpeds.2007.03.020 17719939

[18] Blumenshine P, Egerter S, Barclay CJ, Cubbin C, Braveman PA. Socioeconomic disparities in adverse birth outcomes: a systematic review. Am J Prev Med. 2010;39(3):263–72. 10.1016/j.amepre.2010.05.012 20709259

[19] Ruiz M, Goldblatt P, Morrison J, Kukla L, Švancara J, Riitta-Järvelin M, et al Mother's education and the risk of preterm and small for gestational age birth: a DRIVERS meta-analysis of 12 European cohorts. J Epidemiol Community Health. 2015;69(9):826–33. 10.1136/jech-2014-205387 25911693 PMC4552914

[20] Song YK, Nunez Lopez O, Mehta HB, Bohanon FJ, Rojas-Khalil Y, Bowen-Jallow KA, et al Race and outcomes in gastroschisis repair: a nationwide analysis. J Pediatr Surg. 2017;52(11):1755–1759. 10.1016/j.jpedsurg.2017.03.004 28365103 PMC7772778

[21] Stolar CJ, Crisafi MA, Driscoll YT. Neurocognitive outcome for neonates treated with extracorporeal membrane oxygenation: are infants with congenital diaphragmatic hernia different? J Pediatr Surg. 1995;30(2):366–72 7537811 10.1016/0022-3468(95)90591-x

[22] Dindo D, Demartines N, Clavien PA. Classification of surgical complications: a new proposal with evaluation in a cohort of 6336 patients and results of a survey. Ann Surg. 2004;240(2):205–13. 10.1097/01.sla.0000133083.54934.ae 15273542 PMC1360123

[23] König W, Lüttinger P, Müller W. A comparative analysis of the development and structure of educational systems. Methodological foundations and the construction of a comparative educational scale. CASMIN working paper no. 12. Mannheim: University of Mannheim; 1988.

[24] Braun M, Müller W. Measurement of Education in Comparative Research. Comparative Social Research. 1997,16:163–201.

[25] Brauns H, Steinmann S. Educational Reform in France, West-Germany and the United Kingdom: Updating the CASMIN Educational Classification. 1999: In: ZUMA-Nachrichten, Nr. 44., S. 7–44.

[26] Sagheri D, Hahn P, Hellwig E. The development of a directed population approach to tackle inequalities in dental caries prevalence among secondary school children based on a small area profile. Cent Eur J Public Health. 2008;16(2):65–70. 18661808 10.21101/cejph.a3469

[27] Osler M, Prescott E. Educational level as a contextual and proximate determinant of all cause mortality in Danish adults. J Epidemiol Community Health. 2003 r;57(4):266–9. 10.1136/jech.57.4.266 12646542 PMC1732415

[28] Fiscella K, Franks P. Should years of schooling be used to guide treatment of coronary risk factors? Ann Fam Med. 2004;2(5):469–73. 10.1370/afm.88 15506583 PMC1466706

[29] Müller W, Lüttinger P, König W, Karle W. Class and Education in Industrial Nations. International Journal of Sociology.1989,19: 3–39.

[30] Maxwell S, Brameld K, Bower C, Dickinson JE, Goldblatt J, Hadlow N, et al Socio-demographic disparities in the uptake of prenatal screening and diagnosis in Western Australia. Aust N Z J Obstet Gynaecol. 2011;51(1):9–16. 10.1111/j.1479-828X.2010.01250.x 21299502

[31] Alderdice F, McNeill J, Rowe R, Martin D, Dornan J. Inequalities in the reported offer and uptake of antenatal screening. Public Health. 2008;122(1):42–52. 10.1016/j.puhe.2007.05.004 17645901

[32] Dormandy E, Michie S, Hooper R, Marteau TM. Low uptake of prenatal screening for Down syndrome in minority ethnic groups and socially deprived groups: a reflection of women's attitudes or a failure to facilitate informed choices? Int J Epidemiol. 2005;34(2):346–52. 10.1093/ije/dyi021 15737971

[33] Feijen-de Jong E, Jansen D, Baarveld F, van der Schans C, Schellevis F, Reijneveld S. Determinants of late and/or inadequate use of prenatal healthcare in high-income countries: a systematic review. Eur J Pub Health. 2012;22:904–13. 22109988 10.1093/eurpub/ckr164

[34] Rowe R, Puddicombe D, Hockley C, Redshaw M. Offer and uptake of prenatal screening for Down syndrome in women from different social and ethnic backgrounds. Prenat Diagn. 2008;28(13):1245–50. 10.1002/pd.2125 19039822

[35] Kuppermann M, Learman LA, Gates E, Gregorich SE, Nease RF Jr, Lewis J, et al Beyond race or ethnicity and socioeconomic status: predictors of prenatal testing for Down syndrome. Obstet Gynecol. 2006;107(5):1087–97. 10.1097/01.AOG.0000214953.90248.db 16648415

[36] Khoshnood B, Blondel B, de Vigan C, Bréart G. Socioeconomic barriers to informed decisionmaking regarding maternal serum screening for down syndrome: results of the French National Perinatal Survey of 1998. Am J Public Health. 2004;94(3):484–91. 14998818 10.2105/ajph.94.3.484 PMC1448280

[37] Brindle ME, Flageole H, Wales PW; Canadian Pediatric Surgery Network (CAPSNet). Influence of maternal factors on health outcomes in gastroschisis: a Canadian population-based study. Neonatology. 2012;102(1):45–52. 10.1159/000336564 22507959

[38] Zamakhshary M, Yanchar NL: Complicated gastroschisis and maternal smoking: a causal association? Pediatr Surg Int 2007;23:841–844. 10.1007/s00383-007-1926-6 17618440

[39] Stone ML, Lapar DJ, Kane BJ, Rasmussen SK, McGahren ED, Rodgers BM. The effect of race and gender on pediatric surgical outcomes within the United States. J Pediatr Surg. 2013;48(8):1650–6. 10.1016/j.jpedsurg.2013.01.043 23932602 PMC4219564

[40] Cheng TL, Goodman E; Committee on Pediatric Research. Race, ethnicity, and socioeconomic status in research on child health. Pediatrics. 2015;135(1):e225–37. 10.1542/peds.2014-3109 25548336 PMC9923597

[41] Choo S, Papandria D, Zhang Y, Camp M, Salazar JH, Scholz S, et al Outcomes analysis after percutaneous abdominal drainage and exploratory laparotomy for necrotizing enterocolitis in 4,657 infants. Pediatr Surg Int. 2011;27(7):747–53. 10.1007/s00383-011-2878-4 21400031 PMC4696017

[42] Egbe A, Lee S, Ho D, Uppu S. Effect of Race on the Prevalence of Congenital Malformations among Newborns in the United States. Ethn Dis. 2015;25(2):226–31. 26118153

[43] Lu MC, Halfon N. Racial and ethnic disparities in birth outcomes: a life-course perspective. Matern Child Health J. 2003;7(1):13–30. 12710797 10.1023/a:1022537516969

[44] Vermes G, Mátrai Á, Czeizel AE, Ács N. Maternal factors in the origin of isolated oesophageal atresia: A population-based case-control study. Birth Defects Res A Clin Mol Teratol. 2015;103(9):804–13. 10.1002/bdra.23383 26033843

[45] Chircor L, Mehedinţi R, Hîncu M. Risk factors related to omphalocele and gastroschisis. Rom J Morphol Embryol. 2009;50(4):645–9. 19942960

[46] Oddsberg J, Jia C, Nilsson E, Ye W, Lagergren J. Maternal tobacco smoking, obesity, and low socioeconomic status during early pregnancy in the etiology of esophageal atresia. J Pediatr Surg. 2008;43(10):1791–5. 10.1016/j.jpedsurg.2008.02.058 18926209

[47] Mastroiacovo P. Risk factors for gastroschisis. BMJ. 2008 6 21;336(7658):1386–7. 10.1136/bmj.39577.589699.BE 18558637 PMC2432124

[48] Torfs CP, Velie EM, Oechsli FW, Bateson TF, Curry CJ. A population-based study of gastroschisis: Demographic, pregnancy, and lifestyle risk factors. Teratology 1994;50:44–53. 10.1002/tera.1420500107 7974254

[49] Wilson RD, Johnson MP. Congenital abdominal wall defects: an update. Fetal Diagn Ther. 2004;19(5):385–98. 10.1159/000078990 15305094

[50] Danzer E, Hoffman C, D'Agostino JA, Gerdes M, Bernbaum J, Antiel RM, et al Neurodevelopmental outcomes at 5years of age in congenital diaphragmatic hernia. J Pediatr Surg. 2017;52(3):437–443. 10.1016/j.jpedsurg.2016.08.008 27622588

[51] Wynn J, Aspelund G, Zygmunt A, Stolar CJ, Mychaliska G, Butcher J, et al Developmental outcomes of children with congenital diaphragmatic hernia: a multicenter prospective study. J Pediatr Surg. 2013;48(10):1995–2004. 10.1016/j.jpedsurg.2013.02.041 24094947 PMC3884579

